# Contemporary National Outcomes of Acute Myocardial Infarction-Cardiogenic Shock in Patients with Prior Chronic Kidney Disease and End-Stage Renal Disease

**DOI:** 10.3390/jcm9113702

**Published:** 2020-11-18

**Authors:** Saraschandra Vallabhajosyula, Lina Ya’Qoub, Vinayak Kumar, Dhiran Verghese, Anna V. Subramaniam, Sri Harsha Patlolla, Viral K. Desai, Pranathi R. Sundaragiri, Wisit Cheungpasitporn, Abhishek J. Deshmukh, Kianoush Kashani, Gregory W. Barsness

**Affiliations:** 1 Department of Cardiovascular Medicine, Mayo Clinic, Rochester, MN 55905, USA; Patlolla.SriHarsha@mayo.edu (S.H.P.); Deshmukh.Abhishek@mayo.edu (A.J.D.); Barsness.Gregory@mayo.edu (G.W.B.); 2 Division of Pulmonary and Critical Care Medicine, Department of Medicine, Mayo Clinic, Rochester, MN 55905, USA; kashani.kianoush@mayo.edu; 3 Center for Clinical and Translational Science, Mayo Clinic Graduate School of Biomedical Sciences, Rochester, MN 55905, USA; 4 Section of Interventional Cardiology, Division of Cardiovascular Medicine, Department of Medicine, Emory University School of Medicine, Atlanta, GA 30322, USA; 5 Division of Cardiovascular Medicine, Department of Medicine, Louisiana State University School of Medicine, Shreveport, LA 71115, USA; yaqoublina1989@gmail.com; 6 Department of Medicine, Mayo Clinic, Rochester, MN 55905, USA; kumar.vinayak@mayo.edu (V.K.); subramaniam.anna@mayo.edu (A.V.S.); 7 Department of Medicine, Amita Health Saint Joseph Hospital, Chicago, IL 60657, USA; dhiran.verghese@gmail.com; 8 Department of Medicine, University of Louisville School of Medicine, Louisville, KY 40202, USA; viral.desai@louisville.edu; 9 Division of Hospital Internal Medicine, Department of Medicine, Mayo Clinic, Rochester, MN 55905, USA; drpranathi99@gmail.com; 10 Division of Nephrology and Hypertension, Department of Medicine, Mayo Clinic, Rochester, MN 55905, USA; wcheungpasitporn@gmail.com

**Keywords:** acute myocardial infarction, cardiogenic shock, chronic kidney disease, end-stage renal disease, outcomes research

## Abstract

Background: There are limited data on acute myocardial infarction with cardiogenic shock (AMI-CS) stratified by chronic kidney disease (CKD) stages. Objective: To assess clinical outcomes in AMI-CS stratified by CKD stages. Methods: A retrospective cohort of AMI-CS during 2005–2016 from the National Inpatient Sample was categorized as no CKD, CKD stage-III (CKD-III), CKD stage-IV (CKD-IV) and end-stage renal disease (ESRD). CKD-I/II were excluded. Outcomes included in-hospital mortality, use of coronary angiography, percutaneous coronary intervention (PCI) and mechanical circulatory support (MCS). We also evaluated acute kidney injury (AKI) and acute hemodialysis in non-ESRD admissions. Results: Of 372,412 AMI-CS admissions, CKD-III, CKD-IV and ESRD comprised 20,380 (5.5%), 7367 (2.0%) and 18,109 (4.9%), respectively. Admissions with CKD were, on average, older, of the White race, bearing Medicare insurance, of a lower socioeconomic stratum, with higher comorbidities, and higher rates of acute organ failure. Compared to the cohort without CKD, CKD-III, CKD-IV and ESRD had lower use of coronary angiography (72.7%, 67.1%, 56.9%, 61.1%), PCI (53.7%, 43.8%, 38.4%, 37.6%) and MCS (47.9%, 38.3%, 33.3%, 34.2%), respectively (all *p* < 0.001). AKI and acute hemodialysis use increased with increase in CKD stage (no CKD–38.5%, 2.6%; CKD-III–79.1%, 6.5%; CKD-IV–84.3%, 12.3%; *p* < 0.001). ESRD (adjusted odds ratio [OR] 1.25 [95% confidence interval {CI} 1.21–1.31]; *p* < 0.001), but not CKD-III (OR 0.72 [95% CI 0.69–0.75); *p* < 0.001) or CKD-IV (OR 0.82 [95 CI 0.77–0.87] was predictive of in-hospital mortality. Conclusions: CKD/ESRD is associated with lower use of evidence-based therapies. ESRD was an independent predictor of higher in-hospital mortality in AMI-CS.

## 1. Introduction

Chronic kidney disease (CKD) and end-stage renal disease (ESRD) have been associated with worse clinical outcomes and higher short- and long-term mortality in patients with acute myocardial infarction [AMI] [1,2,3,4]. CKD/ESRD are known predisposing factors for accelerated atherosclerosis and therefore, these patients often present with a higher risk profile and comorbidities, making them more susceptible to acute organ failure, including acute kidney injury (AKI) and mortality [3,5,6,7]. The risk of AKI is increased with an increase in the CKD stage, and AKI itself has been associated with high in-hospital mortality [2,3,8]. As such, it is not surprising that some studies have shown that these patients tend to receive less invasive treatments, including coronary angiography, percutaneous coronary interventions (PCI) and mechanical circulatory support (MCS), mainly due to concerns relating to contrast-associated AKI, worsening kidney function and need for potential acute renal replacement therapy [2,5]. On the other hand, few studies have shown that revascularization following AMI among patients with advanced CKD is associated with improved outcomes and survival.

Traditionally, patients with CKD/ESRD had been excluded in around half of all cardiovascular studies. Thus, clinical outcomes in this high-risk population remain unclear with only limited data and scarce evidence in AMI, especially in cardiogenic shock complicating AMI (AMI-CS) [2,9]. Among a few major trials in AMI-CS, one suggested the prevalence of CKD in AMI-CS to be 17% [10]. A separate analysis of the IABP-SHOCK II (intraaortic balloon pump in cardiogenic shock II) trial showed that serum creatinine >1.5 mg/dL is an independent predictor of 30 day mortality in AMI-CS [11]. Furthermore, prior data have demonstrated that patients with renal insufficiency have higher rates of developing CS [12]. It is known that patients with AMI-CS constitute the sickest spectrum following AMI and have higher comorbidities, worse organ failure and higher in-hospital mortality despite prompt delivery of cardiovascular care [5,6,7,12,13,14,15,16,17,18,19,20,21,22]. There are limited contemporary data from the United States on the outcomes of CKD/ESRD among patients with AMI-CS.

This study sought to evaluate this clinical question using a large national database. We hypothesized that among patients with AMI-CS who have CKD/ESRD, the risk of in-hospital mortality is higher. Furthermore, we hypothesized that admissions with CKD/ESRD would have lower utilization of coronary angiography, PCI and MCS due to their compromised renal function.

## 2. Material and Methods

### 2.1. Study Population, Variables and Outcomes

The National (Nationwide) Inpatient Sample [NIS] is the largest all-payer database of hospital inpatient in the United States. NIS contains discharge data from a 20% stratified sample of community hospitals. The NIS is maintained by the Healthcare Quality and Utilization Project (HCUP), sponsored by the Agency for Healthcare Research and Quality [23]. Information regarding each discharge includes patient demographics, primary payer, hospital characteristics, principal diagnosis, along with up to 39 secondary diagnoses and procedural diagnoses. These data are available to other authors via the HCUP-NIS database with the Agency for Healthcare Research and Quality [23]. Hospital characteristics such as bed size, region and location and teaching status were identified using previously reported methodology [13].

Using the HCUP-NIS data from 2005–2016, a retrospective cohort study of admissions with AMI in the primary diagnosis field (International Classification of Diseases 9.0 Clinical Modification [ICD-9CM] 410.x and ICD-10CM I21.x-22.x) and a secondary diagnosis of CS (ICD-9CM 785.51, ICD-10CM R57.0) were identified [24]. Similar to prior literature, prior CKD/ESRD was identified using ICD-9CM 585.x and ICD-10CM N18.x codes [8]. The use of chronic dialysis was identified using ICD-9CM 39.95/ICD-10CM 5A1D70Z, 5A1D80Z, 5A1D90Z (hemodialysis) and ICD-9CM 54.98/ICD-10CM 3E1M39Z (peritoneal dialysis) in the absence of a concomitant diagnosis of AKI [5,7]. Prior validation studies of CKD administrative codes have shown a sensitivity of 82%, a specificity of 99%, a positive predictive value of 71% and a negative predictive value of 99% [25]. We excluded admissions <18 years of age, with missing in-hospital mortality data, and those with a diagnosis of CKD stage I or stage II since these codes have been found to be insensitive in the hospital setting [26]. The overall AMI-CS cohort was divided into those with no CKD, CKD stage III (CKD-III), CKD stage IV (CKD-IV) and ESRD (CKD-V, ESRD, chronic dialysis) [8]. Deyo’s modification of the Charlson Comorbidity Index was used to identify the burden of comorbid conditions (Supplementary Table S1) [27]. All variables were identified for all admissions using previously used methodologies from our group [5,6,7,12,13,14,15,16,17,19,20,21,28,29,30,31,32,33,34,35,36,37,38]. AKI was identified using ICD-9CM 584 (acute renal failure (ARF), 584.5 (ARF with tubular necrosis), 584.6 (ARF with renal cortical necrosis), 584.7 (ARF with papillary necrosis), 584.8 (ARF with another pathological lesion) and 584.9 (ARF, unspecified), which has been shown to have a high specificity (98%) and negative predictive value (96%) [5].

The primary outcome was the in-hospital mortality in AMI-CS admissions stratified by CKD stage. Secondary outcomes included temporal trends, use of coronary angiography, PCI, MCS, length of stay, hospitalization costs and discharge disposition among the CKD cohorts. We also evaluated the prevalence of AKI and acute hemodialysis use in the no CKD, CKD-III and CKD-IV cohorts.

### 2.2. Statistical Analysis

As recommended by HCUP-NIS, survey procedures using discharge weights provided with the HCUP-NIS database were used to generate national estimates. Using the trend weights provided by the HCUP-NIS, samples from 2005–2011 were re-weighted to adjust for the 2012 HCUP-NIS re-design [39]. One-way analysis of variance and t-tests were used to compare categorical and continuous variables, respectively. Logistic regression was used to analyze trends over time (referent year 2005). The inherent restrictions of the HCUP-NIS database related to research design, data interpretation and data analysis were reviewed and addressed [39]. Pertinent considerations include not assessing individual hospital-level volumes [due to changes to sampling design detailed above], treating each entry as an ‘admission’ as opposed to individual patients, restricting the study details to inpatient factors since the HCUP-NIS does not include outpatient data and limiting administrative codes to those previously validated and used for similar studies [5,6,7,13,14,15,17,19,20,28,29,30,31]. Univariable analyses for trends and outcomes were performed, and their results were represented as odds ratios (OR) with a 95% confidence interval (CI). Multivariable logistic regression analysis incorporating age, sex, race, primary payer status, socioeconomic stratum, hospital characteristics, comorbidities, acute organ failure, AMI-type, cardiac procedures, and noncardiac procedures was performed for temporal trends analyses. For the multivariable modeling, regression analysis with purposeful selection of statistically (liberal threshold of *p* < 0.20 in univariate analysis) and clinically relevant variables was conducted. Two-tailed *p* < 0.05 was considered statistically significant. All statistical analyses were performed using SPSS v25.0 (IBM Corp., Armonk, NY, USA).

## 3. Results

**Figure 1:**
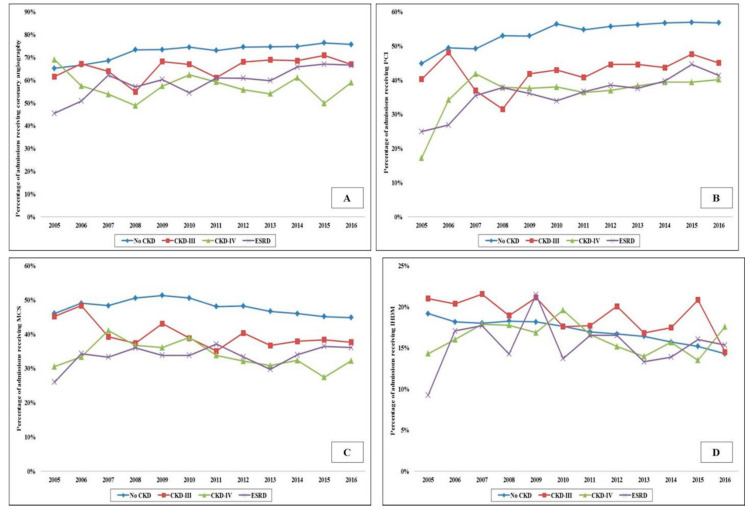
Trends in the use of coronary angiography, PCI, MCS and IHDM, use in AMI-CS admissions stratified by CKD status. Legend: 17 year trends in the use of coronary angiography (**A**), PCI (**B**), MCS (**C**) and IHDM (**D**) in admissions stratified by CKD status; all *p* < 0.001 for trend over time. Abbreviations: AMI: acute myocardial infarction; CKD: chronic kidney disease; CS: cardiogenic shock; ESRD: end-stage renal disease; IHDM: invasive hemodynamic monitoring; MCS: mechanical circulatory support; PCI: percutaneous coronary intervention

**Figure 2:**
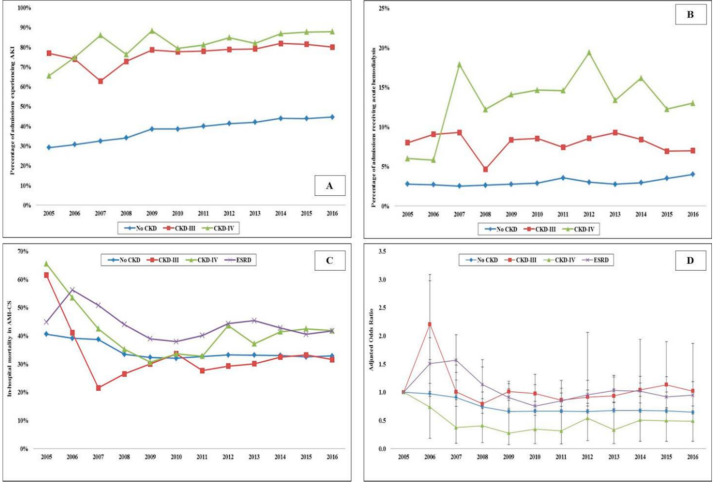
Clinical outcomes in AMI-CS admissions stratified by CKD status. Legend: 17 year unadjusted trends in the prevalence of AKI (**A**) acute hemodialysis; (**B**) use and in-hospital mortality; (**C**) in admissions stratified by CKD status—all *p* < 0.001 for trend over time; (**D)** adjusted multivariate logistic regression for in-hospital mortality temporal trends with 2005 as referent year; adjusted for age, sex, race, comorbidity, primary payer, socioeconomic stratum, hospital characteristics, comorbidities, AMI type, acute organ failure, cardiac arrest, cardiac and noncardiac procedures (*p* < 0.001 for trend over time). Abbreviations: AKI: acute kidney injury; AMI: acute myocardial infarction; CKD: chronic kidney disease; CS: cardiogenic shock; ESRD: end-stage renal disease.

In the period between 1 January 2005, and 31 December 2016, there were over 10 million AMI admissions, of which 372,412 AMI-CS admissions met the inclusion criteria. CKD-III, CKD-IV and ESRD comprised 20,380 (5.5%), 7367 (2.0%) and 18,109 (4.9%), respectively. Admissions with CKD were on average older (69–73 years vs. 68 years), of the White race, bearing Medicare insurance, of a lower socioeconomic stratum, and had higher comorbidities (all *p* < 0.001) (Table 1). The CKD and ESRD cohorts presented more frequently with a non-ST-segment elevation AMI-CS (57–63% vs. 33%), had higher rates of acute organ failure but lower rates of out-of-hospital cardiac arrest (all *p* < 0.001) (Table 1). The use of coronary angiography, PCI, MCS and right heart or pulmonary artery catheterization was lower with higher stages of CKD (Table 1). The temporal trends of coronary angiography, PCI, MCS and invasive hemodynamic monitoring use stratified by CKD stage are presented in Figure 1A–D. The cohorts with CKD and ESRD consistently received less frequent coronary angiography, PCI and MCS over the 12 year study period. The cohorts with no CKD, CKD-III and CKD-IV had progressively higher rates of in-hospital AKI and the use of in-hospital acute hemodialysis (Table 2). The 12 year temporal trends of AKI and acute hemodialysis use in this population are presented in Figure 2A,B. The various CKD stages had consistently higher rates of AKI and acute hemodialysis use during this study period compared to the cohort without CKD.

Compared to those without CKD, the all-cause unadjusted in-hospital mortality was higher in admissions with CKD-IV (34.4% vs. 39.0%; OR 1.22 [95% CI 1.16–1.28]; *p* < 0.001) and ESRD (34.4% vs. 43.2%; OR 1.45 [95% CI 1.41–1.49]; *p* < 0.001), but not CKD-III (34.4% vs. 30.9%; OR 0.85 [95% CI 0.83–0.88]; *p* < 0.001). In a multivariable logistic regression for in-hospital mortality, the cohort with ESRD (OR 1.25 [95% CI 1.21–1.31]; *p* < 0.001), but not CKD-III (OR 0.72 [95% CI 0.69–0.75); *p* < 0.001) or CKD-IV (OR 0.82 [95 CI 0.77–0.87]; *p* < 0.001) had higher in-hospital mortality compared to the cohort without CKD (Supplementary Table S2). The unadjusted and adjusted temporal trends of in-hospital mortality in the cohorts stratified by CKD are presented in Figure 2C,D. There was a steady decrease in in-hospital mortality during this study period, though admissions with CKD-IV and the ESRD cohorts had higher rates than the cohorts with no CKD and CKD-III. The cohorts with CKD and ESRD had a longer hospital stay, higher hospitalization costs and fewer discharges to home (Table 2).

## 4. Discussion

In this nationally representative study, CKD was seen in 12% of all AMI-CS admissions, of which 5% had ESRD. Admissions with CKD were typically older, with higher comorbidities and greater severity of illness. Despite the higher comorbidities and acuity, guideline-directed therapies such as coronary angiography, PCI and MCS were provided less frequently to the CKD and ESRD cohorts. Though temporal trends showed a steady increase in these therapies across all CKD stages, the overall use was lower in comparison with admissions without CKD. ESRD, but not CKD, was an independent marker of higher in-hospital mortality in AMI-CS.

Patients with moderate to severe CKD are considered a high-risk group for accelerated atherosclerosis. This is potentially due to increased inflammation, higher sympathetic tone and increased activity of the renin–angiotensin–aldosterone system [3]. These patients with CKD/ESRD who present with AMI often have high-risk characteristics and more comorbidities, including older age, presence of diabetes mellitus, previous coronary artery disease, left ventricular hypertrophy and congestive heart failure [3]. Our study confirms the higher prevalence of comorbidities, higher incidence of acute organ failure and lower socioeconomic status in patients with CKD/ESRD presenting with AMI-CS. Lower socioeconomic status is associated with worse in-hospital and long-term outcomes and may, therefore, serve both as a cause and effect of advanced comorbidities, including CKD/ESRD [40]. Therefore, CKD patients constitute a higher risk population.

CKD and ESRD have been associated with worse clinical outcomes, including increased cardiovascular complications and mortality in AMI [1,3,41,42,43]. Prior work from our center in 3106 patients with CKD/ESRD and AMI noted incremental in-hospital mortality with higher CKD stages, i.e., 2% in patients with normal renal function, 6% in mild renal failure, 14% in moderate renal failure, 21% in severe kidney failure and 30% in end-stage renal disease (*p* < 0.001) [42]. In a retrospective review of 130,099 patients with CKD, Shlipak et al. found less use of guideline-directed therapies, including PCI, in patients with CKD. They noted a similar trend of incremental mortality with an increase in the CKD stage [43]. In a scientific statement from the American Heart Association in 2003, Sarnak et al. noted long-term mortality is higher in CKD patients after acute coronary syndrome, and the risk of subsequent adverse cardiovascular events is also higher, with a 2 year mortality rate of 50%; double that of patients with normal renal function [4]. In contrast to these prior studies, our study evaluating AMI-CS admissions did not demonstrate CKD to be an independent risk factor for mortality; however, ESRD did predict higher in-hospital mortality. This may be postulated by the following reasons: (a) as compared to those studies, our study population was significantly larger and more representative of the contemporary national practice in the United States; (b) these studies were conducted in the late-1990s and early 2000s when early revascularization was just taking priority in the management of AMI-CS [44]; (c) it is conceivable that with the change in the landscape of AMI-CS, we are treating a sicker population today wherein the higher comorbidity is lesser important that greater illness severity and organ failure [5,6,14,15,17]; (d) careful attention to intra-procedural factors during angiography and careful hemodynamic management of AMI-CS is associated with improvement in clinical outcomes overall [45].

Patients with AMI-CS in the setting of CKD are at increased risk of acute organ failure, including AKI, which itself is known to be associated with worse outcomes [5,7,22,46,47,48]. Furthermore, the receipt of acute dialysis for AKI is associated with an incremental risk over AKI without the need for acute dialysis [22]. In our prior analysis of 440,257 admissions with AMI-CS, we noted AKI and acute dialysis use in 3% and 3.4%, respectively [5]. Compared to admissions without AKI, those with AKI or dialysis requiring AKI were more likely to have prior CKD (7%, 23% and 36%) (*p* < 0.001) [5]. In another retrospective analysis by Marenzi et al., 25% of CS patients with AKI required dialysis, and the use of dialysis was associated with an excess in-hospital mortality risk of 16% compared to those without dialysis (62% versus 46%) [47]. In their Danish registry that included 5079 patients with AMI-CS, Lauridsen et al. found that 13% had AKI requiring dialysis and that these patients had higher in-hospital and long-term mortality over 5 years as well as a higher risk for requiring chronic dialysis [22]. Further data are needed on the optimal angiographic and hemodynamic management of AKI in CKD patients to address this high-risk population.

Patients with AMI-CS in the setting of CKD/ESRD are complex and challenging to manage [1,2,8,9]. It is known that CKD/ESRD patients are at increased risk for both thrombotic and bleeding complications, potentially affecting clinical decision-making regarding the choice of guideline-directed medical therapies, including antithrombotic agents, as well as invasive procedures and reperfusion strategies [1,3,8,41,49]. In addition, patients with CKD are at increased risk for contrast associated-AKI, which itself is associated with increased cardiac complications [3,41]. As such, it is very important to weigh the risks and benefits of management options in patients with CKD/ESRD [5,6,7]. Studies have shown that guideline-directed medical therapies and invasive procedures are less frequently utilized in AMI in patients with CKD/ESRD, despite the fact that patients treated with PCI had lower mortality compared to those treated with conservative medical treatment [1,2,41]. Prior work from our group utilizing the HCUP-NIS database evaluated 4,488,795 hospitalizations for non-ST-segment-elevation AMI and noted CKD or ESRD in 11% [8]. We observed that in non-ST-segment elevation AMI admissions with CKD stages 4 and 5 had 41% and 20% less likelihood, respectively, of undergoing PCI compared with those with no CKD. The cohort treated with PCI had lower in-hospital mortality compared to those managed medically across all CKD stages [8]. Similarly, this current study confirms what prior studies have shown that patients with CKD/ESRD tend to receive less coronary angiography, PCI and MCS. However, as noted above, our study on AMI-CS did not show mortality differences based on the CKD stage as compared to all comers with non-ST-segment elevation AMI.

## 5. Limitations

This study has several limitations, some of which are inherent to the analysis of a large administrative database. The HCUP-NIS attempts to mitigate potential errors by using internal and external quality control measures. The administrative codes for AMI and CS have been previously validated that reduces the inherent errors in the study. Though the administrative codes for CKD have been previously validated, this study does not use laboratory or urine output criteria to determine CKD and AKI, which are more reliable [50,51,52]. The timing of CKD and AKI and treatment-limiting decisions could not be reliably identified in this hospital admissions database. It is possible that a minority of the included admissions had creatinine elevations without AKI [i.e., no tubular injury] [53]. The lack of echocardiographic, hemodynamic and angiographic data, target vessel for PCI, classification, and the presence of multivessel disease, use and doses of inotropes and vasopressors, that may significantly influence outcomes, were not available in this database. Despite the IABP-SHOCK II data demonstrating the limited role of the intra-aortic balloon pump, it remained the most commonly used MCS device in this national study. Despite these limitations, this study addresses an important knowledge gap highlighting the contemporary temporal trends and outcomes of AMI-CS stratified by CKD severity.

## 6. Conclusions

In this national study, prior moderate-to-severe CKD was seen in 12% of all admissions and constituted a vulnerable population with higher comorbidity and acute organ failure. Despite robust guidelines, these populations have lower utilization of coronary angiography, PCI and MCS for AMI-CS management. ESRD, but not CKD, was an independent predictor of higher in-hospital mortality. Further research on the choice of optimal patient, procedural and clinical factors is required to improve clinical outcomes in this sick population.

## Figures and Tables

**Table 1:** Baseline and clinical characteristics of AMI-CS stratified by CKD stage.

Characteristic		No CKD (*N* = 326,556)	CKD-III (*N* = 20,380)	CKD-IV (*N* = 7,367)	ESRD (*N* = 18,109)	*p*
Age (years)		68.1 ± 13.2	73.8 ± 11.3	76.1 ± 10.7	68.5 ± 11.5	<0.001
Female sex		37.2	35.3	40.8	39.3	<0.001
Race	White	66.5	68.9	67.0	49.4	<0.001
	Non-White ^a^	33.5	31.1	33.0	50.6	
Primary payer	Medicare	57.3	76.0	80.3	78.9	<0.001
	Medicaid	7.6	5.5	4.3	5.9	
	Private	25.5	13.8	11.5	12.1	
	Others ^b^	9.5	4.7	3.9	3.1	
Quartile of median household income for zip code	0–25th	27.5	28.2	26.8	31.4	<0.001
	26th–50th	26.8	27.2	28.2	25.2	
	51st–75th	24.5	24.8	24.0	22.9	
	75th–100th	21.2	19.9	20.9	20.5	
Hospital teaching status and location	Rural	6.2	6.4	6.5	3.9	<0.001
	Urban non-teaching	37.1	32.8	33.4	32.5	
	Urban teaching	56.7	60.8	60.1	63.6	
Hospital bed-size	Small	8.5	10.4	10.9	9.1	<0.001
	Medium	23.3	25.1	26.0	22.8	
	Large	68.2	64.4	63.1	68.1	
Hospital region	Northeast	17.8	14.4	16.1	17.4	<0.001
	Midwest	22.8	26.7	23.9	18.6	
	South	38.6	36.9	38.1	40.2	
	West	20.8	22.1	21.9	23.8	
Charlson Comorbidity Index	0–3	28.2	0.8	0.9	3.9	<0.001
	4–6	53.3	24.8	20.2	39.9	
	≥7	18.4	74.4	78.9	56.2	
AMI type	STEMI-CS	66.9	42.7	38.7	37.2	<0.001
	NSTEMI-CS	33.1	57.3	61.3	62.8	
Acute organ failure	Respiratory	48.1	53.7	54.1	52.8	<0.001
	Hepatic	10.7	11.9	12.2	12.1	<0.001
	Hematologic	12.6	16.9	14.6	17.7	<0.001
	Neurologic	16.7	16.2	17.2	20.7	<0.001
Out of hospital cardiac arrest		27.8	20.3	18.1	25.4	<0.001
Coronary angiography		72.7	67.1	56.9	61.1	<0.001
Percutaneous coronary intervention		53.7	43.8	38.4	37.6	<0.001
Coronary artery bypass grafting		17.7	18.6	12.4	16.6	<0.001
Right heart/pulmonary artery catheterization		17.0	17.9	16.6	15.6	<0.001
Mechanical circulatory support	Total	47.9	38.3	33.3	34.2	<0.001
	IABP	45.4	35.1	31.1	30.8	<0.001
	pLVAD	2.9	4.0	2.6	3.7	<0.001
	ECMO	1.0	1.0	0.6	0.9	0.02
Invasive mechanical ventilation		43.6	42.6	43.7	48.4	<0.001
Noninvasive mechanical ventilation		3.8	7.8	8.4	5.5	<0.001

Legend: Represented as percentage or mean ± standard deviation; ^a^ Black, Hispanic, Asian, Native American, others; ^b^ Uninsured, no charge, others. Abbreviations: AMI: acute myocardial infarction; CKD: chronic kidney disease; CS: cardiogenic shock; ECMO: extracorporeal membrane oxygenation; ESRD: end-stage renal disease; IABP: intra-aortic balloon pump; NSTEMI: non-ST-segment elevation myocardial infarction; pLVAD: percutaneous left ventricular assist device; STEMI: ST-segment elevation myocardial infarction.

**Table 2:** Clinical outcomes of AMI-CS stratified by CKD stage.

Outcome		No CKD (*N* = 326,556)	CKD-III (*N* = 20,380)	CKD-IV (*N* = 7367)	ESRD (*N* = 18,109)	*p*
Acute kidney injury		38.5	79.1	84.3	---	<0.001
Acute hemodialysis		2.6	6.5	12.3	---	<0.001
In-hospital mortality		34.4	30.9	39.0	43.2	<0.001
Length of stay (days)		9.6 ± 11.0	10.8 ± 9.9	10.4 ± 9.6	13.6 ± 17.9	<0.001
Hospitalization costs (x1000 USD)		152 ± 177	184 ± 206	160 ± 186	216 ± 305	<0.001
Discharge disposition	Home	43.6	26.2	20.7	24.0	<0.001
	Transferred	10.8	9.5	9.6	10.3	
	Skilled nursing facility	28.2	43.0	47.0	48.1	
	Home with HHC	16.9	20.9	22.4	17.5	
	Against medical advice	0.5	0.4	0.3	0.2	

Legend: Represented as percentage or mean ± standard deviation. Abbreviations: AMI: acute myocardial infarction; CKD: chronic kidney disease; CS: cardiogenic shock; ESRD: end-stage renal disease; HHC: home health care; USD: United States dollars.

## Supplementary Materials

The following are available online at https://www.mdpi.com/2077-0383/9/11/3702/s1. Table S1: Administrative codes used for identification of diagnoses and procedures, Table S2: Predictors of mortality in acute myocardial infarction with cardiogenic shock.

## Abbreviations

AKI	Acute kidney injury
AMI	Acute myocardial infarction
CI	Confidence interval
CKD	Chronic kidney disease
CS	Cardiogenic shock
ESRD	End-stage renal disease
HCUP	Healthcare cost and utilization project
ICD-9CM	International Classification of Diseases-9 Clinical Modification
ICD-10CM	International Classification of Diseases-10 Clinical Modification
MCS	Mechanical circulatory support
NIS	National/Nationwide Inpatient Sample
OR	Odds ratio
PCI	Percutaneous coronary intervention

## References

[1] Santolucito P.A., Tighe D.A., McManus D.D., Yarzebski J., Lessard D., Gore J.M., Goldberg R.J. (2010). Management and outcomes of renal disease and acute myocardial infarction. Am. J. Med..

[2] Hira R.S. (2018). Care of patients with chronic kidney disease presenting with acute coronary syndrome: Improved, but not good enough. J. Am. Heart Assoc..

[3] Han J.H., Chandra A., Mulgund J., Roe M.T., Peterson E.D., Szczech L.A., Patel U., Ohman E.M., Lindsell C.J., Gibler W.B. (2006). Chronic kidney disease in patients with non-ST-segment elevation acute coronary syndromes. Am. J. Med..

[4] Sarnak M.J., Levey A.S., Schoolwerth A.C., Coresh J., Culleton B., Hamm L.L., McCullough P.A., Kasiske B.L., Kelepouris E., Klag M.J. (2003). Kidney disease as a risk factor for development of cardiovascular disease: A statement from the American Heart Association Councils on Kidney in Cardiovascular Disease, High Blood Pressure Research, Clinical Cardiology, and Epidemiology and Prevention. Circulation.

[5] Vallabhajosyula S., Dunlay S.M., Barsness G.W., Vallabhajosyula S., Vallabhajosyula S., Sundaragiri P.R., Gersh B.J., Jaffe A.S., Kashani K. (2019). Temporal trends, predictors, and outcomes of acute kidney injury and hemodialysis use in acute myocardial infarction-related cardiogenic shock. PLoS ONE.

[6] Vallabhajosyula S., Dunlay S.M., Prasad A., Kashani K., Sakhuja A., Gersh B.J., Jaffe A.S., Holmes D.R., Barsness G.W. (2019). Acute noncardiac organ failure in acute myocardial infarction with cardiogenic shock. J. Am. Coll. Cardiol..

[7] Vallabhajosyula S., Ya’Qoub L., Dunlay S.M., Vallabhajosyula S., Vallabhajosyula S., Sundaragiri P.R., Jaffe A.S., Gersh B.J., Kashani K. (2019). Sex disparities in acute kidney injury complicating acute myocardial infarction with cardiogenic shock. ESC Heart Fail..

[8] Bhatia S., Arora S., Bhatia S.M., Al-Hijji M., Reddy Y.N.V., Patel P., Rihal C.S., Gersh B.J., Deshmukh A. (2018). Non-ST-segment-elevation myocardial infarction among patients with chronic kidney disease: A propensity score-matched comparison of percutaneous coronary intervention versus conservative management. J. Am. Heart Assoc..

[9] Januzzi J.L., Cannon C.P., di Battiste P.M., Murphy S., Weintraub W., Braunwald E. (2002). Effects of renal insufficiency on early invasive management in patients with acute coronary syndromes (The TACTICS-TIMI 18 Trial). Am. J. Cardiol..

[10] Alushi B., Douedari A., Froehlig G., Knie W., Wurster T.H., Leistner D.M., Stahli B.E., Mochmann H.C., Pieske B., Landmesser U. (2019). Impella versus IABP in acute myocardial infarction complicated by cardiogenic shock. Open Heart.

[11] Poss J., Koster J., Fuernau G., Eitel I., de Waha S., Ouarrak T., Lassus J., Harjola V.P., Zeymer U., Thiele H. (2017). Risk stratification for patients in cardiogenic shock after acute myocardial infarction. J. Am. Coll. Cardiol..

[12] Vallabhajosyula S., Prasad A., Sandhu G.S., Bell M.R., Gulati R., Eleid M.F., Best P.J.M., Gersh B.J., Singh M., Lerman A. (2019). Mechanical circulatory support-assisted early percutaneous coronary intervention in acute myocardial infarction with cardiogenic shock: 10-year national temporal trends, predictors and outcomes. EuroIntervention.

[13] Vallabhajosyula S., Dunlay S.M., Barsness G.W., Rihal C.S., Holmes D.R., Prasad A. (2019). Hospital-level disparities in the outcomes of acute myocardial infarction with cardiogenic shock. Am. J. Cardiol..

[14] Vallabhajosyula S., Dunlay S.M., Kashani K., Vallabhajosyula S., Vallabhajosyula S., Sundaragiri P.R., Jaffe A.S., Barsness G.W. (2019). Temporal trends and outcomes of prolonged invasive mechanical ventilation and tracheostomy use in acute myocardial infarction with cardiogenic shock in the United States. Int. J. Cardiol..

[15] Vallabhajosyula S., Dunlay S.M., Murphree D.H., Barsness G.W., Sandhu G.S., Lerman A., Prasad A. (2019). Cardiogenic shock in takotsubo cardiomyopathy versus acute myocardial infarction: An 8-year national perspective on clinical characteristics, management, and outcomes. JACC Heart Fail..

[16] Vallabhajosyula S., El Hajj S.C., Bell M.R., Prasad A., Lerman A., Rihal C.S., Holmes D.R., Barsness G.W. (2019). Intravascular ultrasound, optical coherence tomography, and fractional flow reserve use in acute myocardial infarction. Catheter Cardiovasc. Interv..

[17] Vallabhajosyula S., Kashani K., Dunlay S.M., Vallabhajosyula S., Vallabhajosyula S., Sundaragiri P.R., Gersh B.J., Jaffe A.S., Barsness G.W. (2019). Acute respiratory failure and mechanical ventilation in cardiogenic shock complicating acute myocardial infarction in the USA, 2000–2014. Ann. Intensive Care.

[18] Vallabhajosyula S., O’Horo J.C., Antharam P., Ananthaneni S., Vallabhajosyula S., Stulak J.M., Eleid M.F., Dunlay S.M., Gersh B.J., Rihal C.S. (2018). Concomitant intra-aortic balloon pump use in cardiogenic shock requiring veno-arterial extracorporeal membrane oxygenation. Circ. Cardiovasc. Interv..

[19] Vallabhajosyula S., Prasad A., Dunlay S.M., Murphree D.H., Ingram C., Mueller P.S., Gersh B.J., Holmes D.R., Barsness G.W. (2019). Utilization of palliative care for cardiogenic shock complicating acute myocardial infarction: A 15-year national perspective on trends, disparities, predictors, and outcomes. J. Am. Heart Assoc..

[20] Vallabhajosyula S., Prasad A., Gulati R., Barsness G.W. (2019). Contemporary prevalence, trends, and outcomes of coronary chronic total occlusions in acute myocardial infarction with cardiogenic shock. Int. J. Cardiol. Heart Vasc..

[21] Vallabhajosyula S., Vallabhajosyula S., Bell M.R., Prasad A., Singh M., White R.D., Jaffe A.S., Holmes D.R., Jentzer J.C. (2020). Early vs. delayed in-hospital cardiac arrest complicating ST-elevation myocardial infarction receiving primary percutaneous coronary intervention. Resuscitation.

[22] Lauridsen M.D., Gammelager H., Schmidt M., Rasmussen T.B., Shaw R.E., Botker H.E., Sorensen H.T., Christiansen C.F. (2015). Acute kidney injury treated with renal replacement therapy and 5-year mortality after myocardial infarction-related cardiogenic shock: A nationwide population-based cohort study. Crit. Care.

[23] (2009). Introduction to the HCUP Nationwide Inpatient Sample. http://www.hcup-us.ahrq.gov/db/nation/nis/NIS_2009_INTRODUCTION.pdf.

[24] Coloma P.M., Valkhoff V.E., Mazzaglia G., Nielsson M.S., Pedersen L., Molokhia M., Mosseveld M., Morabito P., Schuemie M.J., van der Lei J. (2013). Identification of acute myocardial infarction from electronic healthcare records using different disease coding systems: A validation study in three European countries. BMJ Open.

[25] Quan H., Li B., Saunders L.D., Parsons G.A., Nilsson C.I., Alibhai A., Ghali W.A. (2008). Assessing validity of ICD-9-CM and ICD-10 administrative data in recording clinical conditions in a unique dually coded database. Health Serv. Res..

[26] Ronksley P.E., Tonelli M., Quan H., Manns B.J., James M.T., Clement F.M., Samuel S., Quinn R.R., Ravani P., Brar S.S. (2012). Validating a case definition for chronic kidney disease using administrative data. Nephrol. Dial. Transplant..

[27] Quan H., Sundararajan V., Halfon P., Fong A., Burnand B., Luthi J.C., Saunders L.D., Beck C.A., Feasby T.E., Ghali W.A. (2005). Coding algorithms for defining comorbidities in ICD-9-CM and ICD-10 administrative data. Med. Care.

[28] Subramaniam A.V., Barsness G.W., Vallabhajosyula S., Vallabhajosyula S. (2019). Complications of temporary percutaneous mechanical circulatory support for cardiogenic shock: An appraisal of contemporary literature. Cardiol. Ther..

[29] Vallabhajosyula S., Arora S., Lahewala S., Kumar V., Shantha G.P.S., Jentzer J.C., Stulak J.M., Gersh B.J., Gulati R., Rihal C.S. (2018). Temporary mechanical circulatory support for refractory cardiogenic shock before left ventricular assist device surgery. J. Am. Heart Assoc..

[30] Vallabhajosyula S., Arora S., Sakhuja A., Lahewala S., Kumar V., Shantha G.P.S., Egbe A.C., Stulak J.M., Gersh B.J., Gulati R. (2019). Trends, predictors, and outcomes of temporary mechanical circulatory support for postcardiac surgery cardiogenic shock. Am. J. Cardiol..

[31] Vallabhajosyula S., Deshmukh A.J., Kashani K., Prasad A., Sakhuja A. (2018). Tako-tsubo cardiomyopathy in severe sepsis: Nationwide trends, predictors, and outcomes. J. Am. Heart Assoc..

[32] Vallabhajosyula S., Vallabhajosyula S., Burstein B., Ternus B.W., Sundaragiri P.R., White R.D., Barsness G.W., Jentzer J.C. (2020). Epidemiology of in-hospital cardiac arrest complicating non-ST-segment elevation myocardial infarction receiving early coronary angiography. Am. Heart J..

[33] Vallabhajosyula S., Jentzer J.C., Zack C.J. (2020). Cardiac arrest definition using administrative codes and outcomes in acute myocardial infarction. Mayo Clin. Proc..

[34] Vallabhajosyula S., Kumar V., Vallabhajosyula S., Subramaniam A.V., Patlolla S.H., Verghese D., Ya’Qoub L., Stulak J.M., Sandhu G.S., Prasad A. (2020). Acute myocardial infarction-cardiogenic shock in patients with prior coronary artery bypass grafting: A 16-year national cohort analysis of temporal trends, management and outcomes. Int. J. Cardiol..

[35] Vallabhajosyula S., Patlolla S.H., Dunlay S.M., Prasad A., Bell M.R., Jaffe A.S., Gersh B.J., Rihal C.S., Holmes D.R., Barsness G.W. (2020). Regional variation in the management and outcomes of acute myocardial infarction with cardiogenic shock in the United States. Circ. Heart Fail..

[36] Vallabhajosyula S., Bell M.R., Sandhu G.S., Jaffe A.S., Holmes D.R., Barsness G.W. (2020). Complications in patients with acute myocardial infarction supported with extracorporeal membrane oxygenation. J. Clin. Med..

[37] Vallabhajosyula S., Shankar A., Patlolla S.H., Prasad A., Bell M.R., Jentzer J.C., Arora S., Vallabhajosyula S., Gersh B.J., Jaffe A.S. (2020). Pulmonary artery catheter use in acute myocardial infarction-cardiogenic shock. ESC Heart Fail..

[38] Vallabhajosyula S., Patlolla S.H., Verghese D., Ya’Qoub L., Kumar V., Subramaniam A.V., Cheungpasitporn W., Sundaragiri P.R., Noseworthy P.A., Mulpuru S.K. (2020). Burden of arrhythmias in acute myocardial infarction complicated by cardiogenic shock. Am. J. Cardiol..

[39] Khera R., Krumholz H.M. (2017). With great power comes great responsibility: Big data research from the National Inpatient Sample. Circ. Cardiovasc. Qual. Outcomes.

[40] Schultz W.M., Kelli H.M., Lisko J.C., Varghese T., Shen J., Sandesara P., Quyyumi A.A., Taylor H.A., Gulati M., Harold J.G. (2018). Socioeconomic status and cardiovascular outcomes: Challenges and interventions. Circulation.

[41] Conti C.R. (2003). Management of patients with acute myocardial infarction and end-stage renal disease. J. Am. Coll. Cardiol..

[42] Wright R.S., Reeder G.S., Herzog C.A., Albright R.C., Williams B.A., Dvorak D.L., Miller W.L., Murphy J.G., Kopecky S.L., Jaffe A.S. (2002). Acute myocardial infarction and renal dysfunction: A high-risk combination. Ann. Intern. Med..

[43] Shlipak M.G., Heidenreich P.A., Noguchi H., Chertow G.M., Browner W.S., McClellan M.B. (2002). Association of renal insufficiency with treatment and outcomes after myocardial infarction in elderly patients. Ann. Intern. Med..

[44] Hochman J.S., Sleeper L.A., Webb J.G., Sanborn T.A., White H.D., Talley J.D., Buller C.E., Jacobs A.K., Slater J.N., Col J. (1999). Early revascularization in acute myocardial infarction complicated by cardiogenic shock. SHOCK investigators. Should we emergently revascularize occluded coronaries for cardiogenic shock. N. Engl. J. Med..

[45] Vallabhajosyula S., Barsness G.W., Vallabhajosyula S. (2019). Multidisciplinary teams for cardiogenic shock. Aging (Albany NY).

[46] Fox C.S., Muntner P., Chen A.Y., Alexander K.P., Roe M.T., Wiviott S.D. (2012). Short-term outcomes of acute myocardial infarction in patients with acute kidney injury: A report from the national cardiovascular data registry. Circulation.

[47] Marenzi G., Assanelli E., Campodonico J., de Metrio M., Lauri G., Marana I., Moltrasio M., Rubino M., Veglia F., Montorsi P. (2010). Acute kidney injury in ST-segment elevation acute myocardial infarction complicated by cardiogenic shock at admission. Crit. Care Med..

[48] Sun Y.B., Tao Y., Yang M. (2018). Assessing the influence of acute kidney injury on the mortality in patients with acute myocardial infarction: A clinical trial. Ren. Fail..

[49] Smilowitz N.R., Gupta N., Guo Y., Mauricio R., Bangalore S. (2017). Management and outcomes of acute myocardial infarction in patients with chronic kidney disease. Int. J. Cardiol..

[50] Kotecha A., Vallabhajosyula S., Coville H.H., Kashani K. (2018). Cardiorenal syndrome in sepsis: A narrative review. J. Crit. Care.

[51] Sakhuja A., Bandak G., Barreto E.F., Vallabhajosyula S., Jentzer J., Albright R., Kashani K.B. (2019). Role of loop diuretic challenge in stage 3 acute kidney injury. Mayo Clin. Proc..

[52] Vallabhajosyula S., Sakhuja A., Geske J.B., Kumar M., Kashyap R., Kashani K., Jentzer J.C. (2018). Clinical profile and outcomes of acute cardiorenal syndrome type-5 in sepsis: An eight-year cohort study. PLoS ONE.

[53] Ahmad T., Jackson K., Rao V.S., Tang W.H.W., Brisco-Bacik M.A., Chen H.H., Felker G.M., Hernandez A.F., O’Connor C.M., Sabbisetti V.S. (2018). Worsening renal function in patients with acute heart failure undergoing aggressive diuresis is not associated with tubular injury. Circulation.

