# Supplementary material for: Contemporary National Outcomes of Acute Myocardial Infarction-Cardiogenic Shock in Patients with Prior Chronic Kidney Disease and End-Stage Renal Disease
Source: J Clin Med. 2020 Nov 18;9(11):3702. doi: 10.3390/jcm9113702 (PMC7698908; doi:10.3390/jcm9113702)
Supplement: Supplementary file 1 [file jcm-09-03702-s001.zip › jcm-989742-supplementary.docx]

**Supplementary Table S1. Administrative codes used for identification of diagnoses and procedures**

| **Comorbidity** | **International Classification of Diseases, 9.0 Clinical Modification codes** |
| --- | --- |
| Cardiac arrest | 427.5 |
| Cardiogenic shock | 785.51 |
| Acute kidney injury | 584, 584.5, 584.6, 584.7, 584.8, 584.9 |
| Respiratory failure | 518.81, 518.82, 518.85, 786.09, 799.1, 96.7, 96.70, 96.71, 96.72 |
| Hepatic failure | 570.0, 572.2, 573.3, 573.4 |
| Hematologic failure | 286.6-286.9, 287.4, 287.5 |
| Neurological failure | 293, 293.0, 293.1, 293.8, 293.81-293.84, 293.89, 293.9, 348.1, 348.3, 348.30, 348.81, 348.39, 780.01, 780.09, 89.14 |
| Invasive hemodynamic assessment | 37.21, 37.23, 204 |
| Coronary angiography | 37.22, 37.23, 88.53-88.56 |
| Percutaneous coronary intervention | 00.66, 36.01, 36.02, 36.05, 36.06, 36.07, 88.57 |
| Intra-aortic balloon pump | 37.61 |
| Percutaneous mechanical circulatory support | 37.68 |
| Extra-corporeal membrane oxygenation | 39.65 |
| Invasive mechanical ventilation | 96.7, 96.70, 96.71, 96.72 |

**Supplementary Table S2. Predictors of mortality in acute myocardial infarction with cardiogenic shock**

| **Total cohort (N = 372,412)** | | **Odds ratio** | **95% confidence interval** | | ***P*** |
| --- | --- | --- | --- | --- | --- |
|  |  |  | **Lower Limit** | **Upper Limit** |  |
| **No chronic kidney disease** | | Reference category | | | |
| **Chronic kidney disease, stage III** | | 0.72 | 0.69 | 0.75 | <0.001 |
| **Chronic kidney disease, stage IV** | | 0.82 | 0.77 | 0.87 | <0.001 |
| **End-stage renal disease** | | 1.25 | 1.21 | 1.31 | <0.001 |
| **Age groups (years)** | **≤75 years** | Reference category | | | |
|  | **>75 years** | 1.91 | 1.86 | 1.95 | <0.001 |
| **Female sex** | | 1.09 | 1.07 | 1.11 | <0.001 |
| **Race** | **White** | Reference category | | | |
|  | **Black** | 0.92 | 0.89 | 0.95 | <0.001 |
|  | **Hispanic** | 1.00 | 0.97 | 1.03 | 0.99 |
|  | **Asian/Pacific Islander** | 0.94 | 0.90 | 0.99 | 0.02 |
|  | **Native American** | 1.01 | 0.91 | 1.13 | 0.85 |
|  | **Other** | 0.98 | 0.94 | 1.03 | 0.45 |
| **Primary payer** | **Medicare** | Reference category | | | |
|  | **Medicaid** | 0.74 | 0.71 | 0.77 | <0.001 |
|  | **Private** | 0.70 | 0.68 | 0.72 | <0.001 |
|  | **Uninsured** | 1.13 | 1.08 | 1.17 | <0.001 |
|  | **No Charge** | 0.83 | 0.73 | 0.95 | 0.005 |
|  | **Others** | 0.82 | 0.77 | 0.87 | <0.001 |
| **Quartile of median household**  **income for zip code** | **0-25^th^** | Reference category | | | |
|  | **26^th^-50^th^** | 0.95 | 0.93 | 0.97 | <0.001 |
|  | **51^st^-75^th^** | 0.93 | 0.91 | 0.96 | <0.001 |
|  | **75^th^-100^th^** | 0.87 | 0.85 | 0.89 | <0.001 |
| **Hospital teaching**  **status and location** | **Rural** | Reference category | | | |
|  | **Urban Non-Teaching** | 0.99 | 0.95 | 1.03 | 0.53 |
|  | **Urban Teaching** | 1.05 | 1.01 | 1.09 | 0.01 |
| **Hospital bed-size** | **Small** | Reference category | | | |
|  | **Medium** | 1.01 | 0.97 | 1.04 | 0.61 |
|  | **Large** | 1.04 | 1.01 | 1.08 | 0.006 |
| **Hospital region** | **Northeast** | Reference category | | | |
|  | **Midwest** | 1.04 | 1.01 | 1.07 | 0.02 |
|  | **South** | 1.10 | 1.08 | 1.13 | <0.001 |
|  | **West** | 1.01 | 0.98 | 1.04 | 0.61 |
| **Year of admission** | **2005** | Reference category | | | |
|  | **2006** | 1.00 | 0.96 | 1.05 | 0.88 |
|  | **2007** | 0.94 | 0.89 | 0.98 | 0.004 |
|  | **2008** | 0.76 | 0.72 | 0.79 | <0.001 |
|  | **2009** | 0.67 | 0.64 | 0.70 | <0.001 |
|  | **2010** | 0.67 | 0.64 | 0.70 | <0.001 |
|  | **2011** | 0.67 | 0.64 | 0.70 | <0.001 |
|  | **2012** | 0.68 | 0.66 | 0.71 | <0.001 |
|  | **2013** | 0.70 | 0.67 | 0.73 | <0.001 |
|  | **2014** | 0.70 | 0.67 | 0.73 | <0.001 |
|  | **2015** | 0.70 | 0.67 | 0.73 | <0.001 |
|  | **2016** | 0.68 | 0.65 | 0.71 | <0.001 |
| **Charlson Comorbidity Index** | **0-3** | Reference category | | | |
|  | **4-6** | 1.13 | 1.10 | 1.16 | <0.001 |
|  | **≥ 7** | 1.15 | 1.11 | 1.19 | <0.001 |
| **Acute organ dysfunction** | **Respiratory** | 1.25 | 1.22 | 1.27 | <0.001 |
|  | **Hepatic** | 1.65 | 1.61 | 1.70 | <0.001 |
|  | **Hematologic** | 0.95 | 0.93 | 0.98 | 0.001 |
|  | **Neurological** | 1.62 | 1.58 | 1.66 | <0.001 |
| **Coronary angiography** | | 0.54 | 0.53 | 0.55 | <0.001 |
| **Percutaneous coronary intervention** | | 0.57 | 0.56 | 0.58 | <0.001 |
| **Coronary artery bypass grafting** | | 0.27 | 0.26 | 0.28 | <0.001 |
| **Invasive hemodynamic monitoring** | | 0.96 | 0.94 | 0.99 | 0.002 |
| **Mechanical circulatory support** | | 1.37 | 1.34 | 1.40 | <0.001 |
| **Invasive mechanical ventilation** | | 2.12 | 2.08 | 2.16 | <0.001 |
| **Non-invasive ventilation** | | 0.65 | 0.62 | 0.68 | <0.001 |
